# Advanced Roux-en-Y hepaticojejunostomy with magnetic compressive anastomats in obstructive jaundice dog models

**DOI:** 10.1007/s00464-017-5740-5

**Published:** 2017-08-04

**Authors:** Chao Fan, Hongke Zhang, Xiaopeng Yan, Jia Ma, Chunbao Wang, Yi Lv

**Affiliations:** 1 Department of Hepatobiliary Surgery, Institute of Advanced Surgical Technology and Engineering, Shaanxi Center for Regenerative Medicine and Surgical Engineering, The First Affiliated Hospital of Xi’an Jiaotong University, Xi’an, 710061 Shaanxi China; 2 Department of Surgical Oncology, Shaanxi Province People’s Hospital, Xi’an, Shaanxi China; 3 Department of Pathology, The First Affiliated Hospital of Xi’an Jiaotong University, Xi’an, Shaanxi China; 4 Department of Hepatobiliary Surgery, Medical School of Xi’an Jiaotong University, Xi’an, 710061 Shaanxi China

**Keywords:** Hepaticojejunostomy, Bilioenteric anastomosis, Anastomat, Obstructive jaundice, Sutureless, Magnetic compression anastomosis, Wound healing

## Abstract

**Background:**

Although commonly used procedure, Roux-en-Y hepaticojejunostomy (RYHJ) remains to be complicated, time consuming, and has a relatively poor prognosis. We designed the magnetic compressive anastomats (MCAs) to perform RYHJ more efficiently and safely.

**Materials and methods:**

36 dogs were divided into two groups randomly. After obstructive jaundice model construction, RYHJ was performed with MCAs in study group or by hand-sewn in control group. Both groups were followed for 1, 3, and 6 months after RYHJ. The liver function and postoperative complications were recorded throughout the follow-up. At the end of each time point, dogs were sent for magnetic resonance imaging (MRI) and sacrificed. Anastomotic samples were taken for anastomotic narrowing rate calculation, histological analyses, tensile strength testing, and hydroxyproline content testing.

**Results:**

The anastomotic construction times were 44.20 ± 23.02 min in study group, compared of 60.53 ± 11.89 min in control group (*p* < 0.05). The liver function recovered gradually after RYHJ in both groups (*p* > 0.05). All anastomats were expelled out of the body in 8.81 ± 2.01 days. The gross incidence of morbidity and mortality was 33.3% (6/18) and 16.7% (3/18) in study group compared with 38.9% (7/18) and 22.2% (4/18) in control group (*p* > 0.05), and there is no single case of anastomotic-specific complications happened in study group. The narrowing rates of anastomosis were 14.6, 18.5, and 18.7% in study group compared with 35.4, 36.9, and 34% in control group at 1st, 3rd, and 6th month after RYHJ (*p* < 0.05). In study group, preciser alignment of tissue layers and milder inflammatory reaction contributed to the fast and better wound healing process.

**Conclusion:**

Perform RYHJ with MCAs is safer, more efficient than by hand-sewn method in obstructive jaundice dog models.

**Electronic supplementary material:**

The online version of this article (doi:10.1007/s00464-017-5740-5) contains supplementary material, which is available to authorized users.

Roux-en-Y hepaticojejunostomy (RYHJ) is a common procedure for bypassing hepatic biliary obstructions and establishing bilioenteric continuity after resections for various disease and injuries [1, 2]. However, it is always a technical challenge to construct multiple bilioenteric anastomoses in limited operative field, and the following postoperative complications, such as anastomotic stricture and bile leakage, would influence the prognosis of the patients [3, 4]. Up to now, how to perform RYHJ efficiently and safely remains to be a problem.

The magnetic compressive anastomosis (MCA) was first reported in 1978 [5]. Its working principle is based on that the magnetic compression force leads to gradual tissue necrosis within magnets while tissue healing at the edge of the magnet simultaneously [6]. Although the MCA belongs to the mechanical compression anastomosis family similar to “Murphy’s Button [7],” “The Valtrac Biofragmentable Anastomotic Ring (BAR) [8],” or “Compression Anastomosis Clip (CAC) [9],” the underlying compressive force is direct coming from the attraction of magnets rather than complicated mechanical structure like buckle. Therefore, the structure of MCA is simple and the compressive strength on tissue is continuous and changeable throughout the anastomotic process (the strength between the magnets keeps increasing while the compressed tissue is getting thinner and the distance between the magnets becomes closer). With its unique characters, the MCA has been used to construct different hollow viscera anastomosis, such as esophagus [10], gastrointestinal tract [6, 11–13], bile duct [14], and vascular [15, 16], to improve the results of the surgery. It is considered to be a safe surgical technique that is equivalent or superior to anastomoses created by traditional sutures or stapling techniques [10]. For certain diseases (bile duct anastomotic stricture or esophageal atresia) [17–20] or under extreme circumstances (inflammatory or infectious situation) [21–23], the MCA was as the safest way to construct anastomosis.

With more than 10 years’ study on magnamosis [21, 24–27], we got a lot of experiences on how to overcome the limitations of magnets, such as combine different materials with magnet to improve the machinability of MCA; add electroplate layers on magnets against corrosion in moisture environment of the body; coat static magnetic field shielders on magnets to keep the isolation of MCA from other paramagnets; develop nano-composite permanent magnet materials to make the vascular MCA device biodegradable. Through these years, we try to broaden the magnamosis on surgical applications and figure out how magnetic force influences the wound healing process.

Herein, we designed a set of magnetic compressive anastomats (including bilioenteric anastomats and enteroenteric anastomats) to facilitate the RYHJ, reduce the postoperative complications, and improve the prognosis in obstructive jaundice model of dog.

## Methods

### The type of the study

Randomized animal/observational experiment was performed in the study.

### The biliary-enteric anastomats

**Fig. 1:**
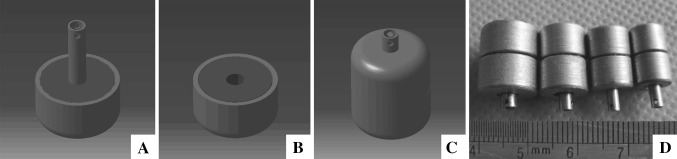
The design drawings and device of the BE-MCAs. **A** Mother part of BE-MCAs. **B** Daughter part of BE-MCAs. **C** Coupled BE-MCAs. **D** Real anastomats in different sizes

The anastomats were designed as shown in Fig. 1, each anastomat included a mother part (Fig. 1A) and a daughter part (Fig. 1B), and both of which consisted magnetic ring core and static magnetic field shielder (SMS). The magnetic rings were made of sintered-type neodymium-iron-boron (NdFeB, N52), and the SMSs were made of electrical pure iron. Both NdFeB ring and SMS were plated a titanium-nitride layer to improve erosion resistance and biocompatibility in body. There were two side holes at the end of the center duct of the SMS. Four sizes of MCAs were produced for bilioenteric anastomosis construction (Fig. 1D); the outer diameters (OD) of the MCAs were 10.5, 8.5, 6.5, and 5.5 mm, respectively (Fig. 1D).

The enteroenteric anastomats used in this study were mentioned before [28].

### The animals and grouping

Thirty-six adult hybrid dogs weighing around 15 kg were provided by the Experimental Animal Center, Medical College of Xi’an Jiaotong University. All dogs received humane care in compliance with the Guide for the Care and Use of Laboratory Animals published by the National Institutes of Health, and the protocol was approved by the Institutional Animal Care and Use Committee of Medical College. The experimental conducting and paper submission was approved by The Human and Ethical Committee for Medical Research at Xi’an Jiaotong University College of Medicine.

These dogs were randomly divided into two groups (18 for each group): the study group and the control group. After a common bile duct (CBD) ligation for 7–10 days, the dogs were performed RYHJ with MCAs in study group or by hand-sewn in control group. Both groups were then divided into three subgroups for following observation, and the follow-up periods were 1, 3, and 6 months, respectively.

### Dog anatomy and RYHJ procedure

Obstructive jaundice model was constructed by common bile duct ligation in 7–10 days as we described before [28]. All dogs had seven liver lobes; the extrahepatic bile ducts distributed for 2–4 branches, and 64% (23/36) of them showed 3 branches (right, middle, and left) before converging to form common bile duct. No differences were found regarding the constituent ratio between two groups for number of extrahepatic bile ducts (*p* > 0.582). At the time for RYHJ, the OD of the hepatic bile duct dilated to 5.51 ± 3.45, 11.01 ± 3.90, and 7.55 ± 3.17 mm from right to left through abdominal B-ultrasound scanning.

Trachea intubation and assistant respiration were initiated after general anesthesia, and continuous venous intransfusion was provided throughout the surgery. The abdomen was incised along the previous surgical scar. Hepatic hilar was exposed and the extrahepatic bile ducts were cut transversely above convergence of CBD. The inner diameter (ID) of biliary stumps was measured with an electronic vernier caliper. The extrahepatic bile duct, in which ID was less than 5.5 mm, was ligated directly in both groups, and the suitable-sized anastomats were chosen for anastomotic construction in study group. The jejunum was transected at 20-cm distal from the treitz ligament for later procedures.

In control group, the stump of the distal jejunum was closed, and the loop was mobilized to porta hepatis for multiple, single layer interrupted end-to-side hepaticojejunostomy as described by Blumgart [29]. The proximal stump was performed an end-to-side anastomosis with the jejunum at distal 50 cm from the transected side.

**Fig. 2:**
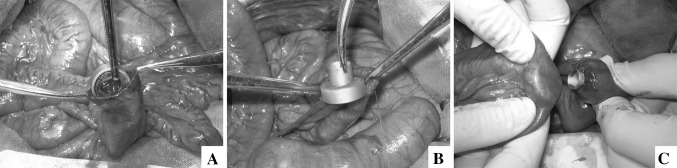
Enteroenterostomy with MCAs. **A** The daughter part of EE-MCAs was put in from the opening of distal jejunum. **B** The mother part of EE-MCAs was fixed at proximal end of jejunum. **C** Two parts of EE-MCAs were coupled together

**Fig. 3:**
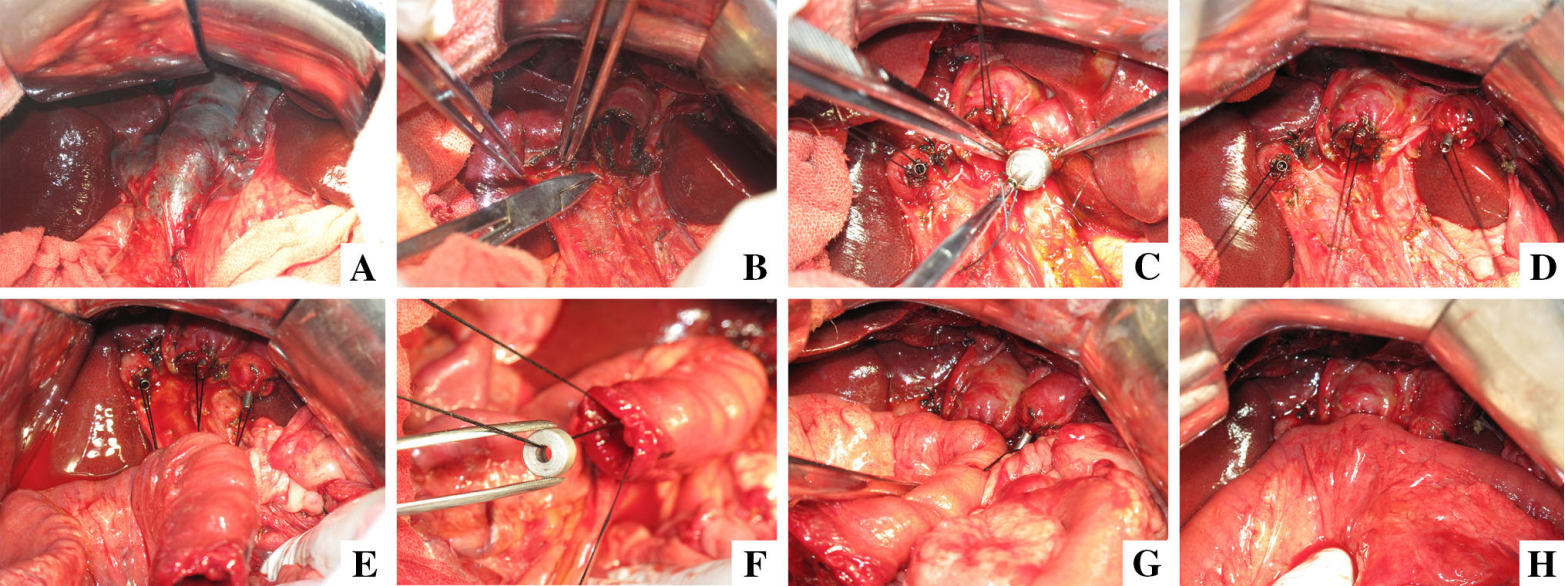
Hepaticojejunostomy with MCAs. **A** Extrahepatic bile ducts dilated obviously after BDL. **B** Purse-string suturing at the end of biliary stumps. **C** The mother parts of BE-MCAs were inserted into the bile duct and fixed at the end of stumps successively. **D** Three mother parts of MCAs were fixed at hepatic duct stumps. **E** All guide strings passed through the jejunal wall. **F** Corresponding daughter part approximated to the porta hepatis through guidance. **G** The right and middle biliary-enteric anastomoses were constructed successfully. **H** Finished three anastomoses with MCAs

In study group, all of the anastomoses were constructed with MCAs. The stump of the distal jejunum was kept opening at the beginning. The daughter part of enteroenteric magnetic compression anastomats (EE-MCAs) was put in the opening of distal jejunum (Fig. 2A) and slided to distal 50 cm along the jejunal lumen. The mother part of EE-MCAs was introduced and fixed (Fig. 2B) at purse-string-sutured proximal end of jejunum. Then, pressed daughter part against mother part, the intestine wall on mother part was punched by central duct of the daughter part and two parts were coupled together (Fig. 2C). After enteroenteric anastomosis (EEA), all biliary stumps were purse-string sutured with 4–0 Prolene^®^ sequentially (ETHICON; Johnson & Johnson, Somerville, New Jersey, USA) (Fig. 3B). Then each mother part of bilioenteric magnetic compression anastomats (BE-MCAs) was passed through a 3–0 silk suture (ETHICON; Johnson & Johnson, Somerville, New Jersey, USA) from side holes at the end of the central duct, and was introduced and fixed at the stumps of the bile ducts (Fig. 3C, D). Three holes were punched on jejunal wall at 5 cm away from the jejunal opening. The silk sutures passed through the holes from outside to enteric lumen and were pulled out of the jejunal opening (Fig. 3E). The corresponding daughter parts were acrossed through the silk sutures and slided forward to the porta hepatis, and coupled with the mother parts from right to left (Fig. 2F–H). Finally, all guide silk sutures were drawn off and the opening of the jejunal stump was closed. Washed the abdominal cavity and closed the abdomen.

### Anastomotic construction time

The times for exact multiple bilioenteric anastomosis construction were recorded during the surgery in dogs and compared between the groups.

### Post-operative follow-up

All dogs were monitored carefully until fully recovered from the anesthesia after operation. Water was available at the 2nd day and solid foods were provided at the 3rd day after RYHJ. 500–1000 mL of Lactated Ringer’s Solution was intravenously resuscitated daily until the animal recovered appetite. The drainage tube was removed when the volume of abdominal fluid was less than 10 mL per day. Appropriate treatment, such as exploratory laparotomy or anti-inflammatory therapies would be done if the drainage fluid were abnormal.

### Liver function test (LFT)

Liver function was routinely tested perioperatively for all dogs. The time points for blood sample collection were pre-CBD ligation, pre-RYHJ, and 1, 2, 3rd week, 1, 2, 3, 4, 5, 6th month after RYHJ.

### Imaging examination

In study group, instant abdominal fluoroscopy was performed after the RYHJ to confirm the successful coupling of the anastomats, and subsequent daily plain film was taken to check the expelling of anastomats through the intestine.

### Postoperative complications

The golden standard for diagnosis was pathological observation. The dogs were performed autopsy once died or at the end of time-points. Bile leakage was highly suspected when dog got anorexia, vomiting, shivering and yellowish-brown ascites flowing out from scar. The anastomotic stricture was doubted when TBIL value was elevated or kept in abnormal high after steady decline, or by characteristic abdominal MRI scan.

### Specimen harvest, histological analyses, and wound healing evaluation

At the end of each time point, the dogs were sacrificed by overdosed high concentration of carbon dioxide (CO_2_) respiration. All anastomoses were harvested for wound healing evaluation. The ID of the anastomosis was measured by electronic vernier caliper again after gross observation. The total ID of each dog, which was the sum of IDs of all extrahepatic bile ducts, was calculated for later comparison. The initial ID of bile duct stumps in study group was equal to the OD of used anastomats.

Then all anastomotic specimens were cut to three parts along the anastomotic line. One part was fixed in 10% buffered formalin solution for hematoxylin and eosin (H&E) staining. One part was immersed in 4 °C University of Wisconsin (UW) Solution for instant tensile strength testing. The last part was kept in −80 °C freezer for hydroxyproline content testing.

The anastomotic healing was evaluated by semiquantitative histological analysis which was modified method of Attard [30] and Biert [31] (Table 1). For each histologic section of anastomotic specimen, seven wound healing-related histological parameters were ranked from 0 to 3 points by pathologist, and the scores of specimens at same time point were compared between the groups. The same pathologist who was blinded to the study observed all slides.

**Table 1:** Histological parameters and scoring for anastomotic healing

Histological parameters	Score criteria for judging			
	0 = None	1 = Poor	2 = Good	3 = Excellent
Bridging				
Mucosal continuity				
Muscular continuity				
Reepithelialization				

	0 = Massive	1 = Marked increase	2 = Slight increase	3 = Normal
Inflammatory reaction				
Polymorph nuclear cells				
Lymphocytes				

	0 = Massive	1 = Some patches	2 = Small patch	3 = None
Necrosis				
Edema				

Tensile strength of BE anastomoses was tested by electronic universal testing machine (CMT6503, SANS). All anastomotic specimens were prepared as rectangle strips with length above 2 cm and width above 0.5 cm. The exact width of each samples was measured by electronic vernier caliper, and results were calculated as follows: Tensile Strength = Fmax (N)/width (mm).

Tissue samples of 0.2 g wet weight were used to test anastomotic hydroxyproline concentrations at different time-points by chemical colorimetry with hydroxyproline test box (Fuzhou MaiXin Biotechnology Development Co., Ltd).

### Statistical analyses

The spss statistics 17.0 software was used for data analysis. The quantitative results were expressed as mean ± standard deviation (SD). Differences between groups were analyzed by the independent-samples *T* test, Mann–Whitney *U* Test, analysis of variance (ANOVA), and Fisher’s exact test. Significant difference was considered if *p* < 0.05.

## Results

### Anastomotic construction time

The anastomotic construction times were 44.20 ± 23.02 and 60.53 ± 11.89 min in the MCAs group and the hand-sewn group, respectively (*p* < 0.05).

### Liver function test

For both groups, ALP, ALT, and DBIL increased dramatically after common bile duct ligation and decreased sharply in 1st week after RYHJ. Then ALP and ALT fluctuated at relative higher level in following period. The DBIL, however, decreased to normal level between 2nd and 3rd month after RYHJ. The LFT at each time point between two groups had no difference (*p* > 0.05).

### The expelling process and the expulsion time of MCAs

**Fig. 4:**
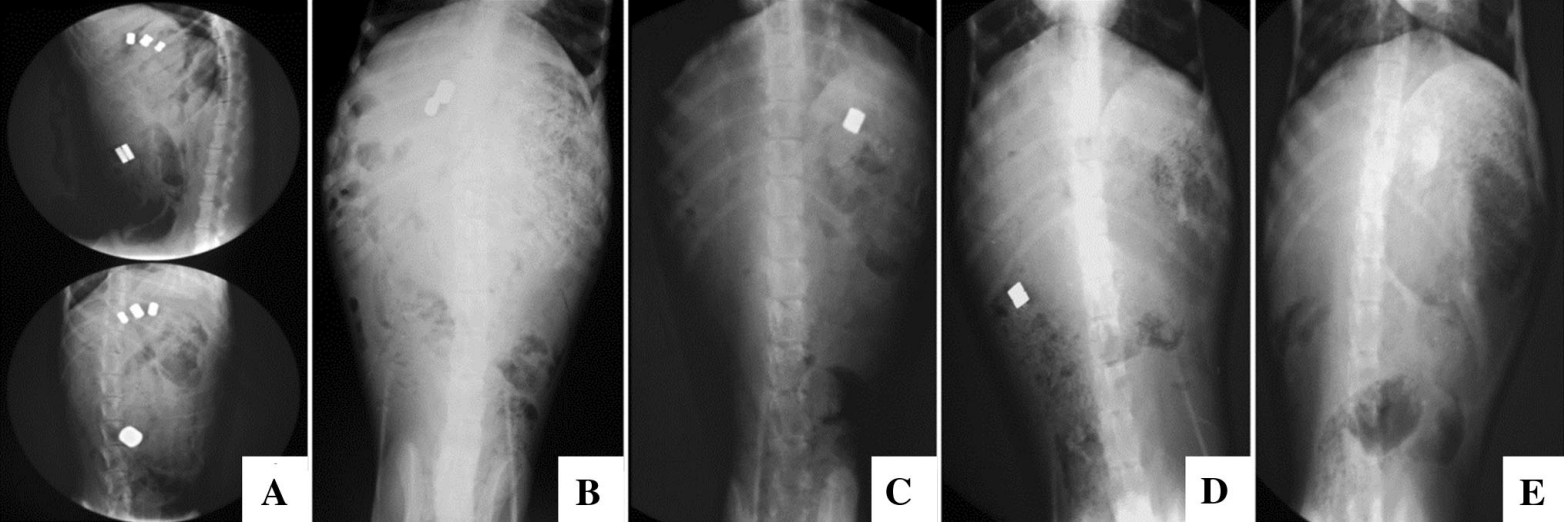
The expelling process of MCAs was monitored by X-ray. **A** The instant fluoroscopy showed all anastomats coupled well and retained at the right place (*upper* lateral view; *lower* anteroposterior view). **B** At 4th day after RYHJ, EE-MCA and the first BE-MCA were expelled out of the body, and two BE-MCAs remained in situ. **C** At 8th day after RYHJ, the second BE-MCA was missing, and the third one already dropped in small intestine. **D** At 9th day after RYHJ, the third BE-MCA was expelled at hepatic flexure of ascending colon. **E** At 10th day, all anastomats were expelled out of the body

The anastomats in 17 dogs were expelled out of the body successively in 2 weeks (Fig. 4A–E), and the average expulsion time was 8.81 ± 2.01 days. Except for one dog, three BE-MCAs were failure to drop off because two parts of anastomat did not couple precisely, and the leaked magnetic field made these BE-MCAs attracted together.

### Postoperative complications and anastomotic narrowing rates

The incidence of morbidity and mortality were 38.9% (7/18) and 22.2% (4/18) in hand-sewn group, compared of 33.3% (6/18) and 16.7% (3/18) in MCAs group (*p* > 0.05). The postoperative complications of RYHJ in both groups are detailed in Table 2. The anastomat application faults in study group were showed in supplementary materials (Table 5, Figs. 9, 10). The narrowing rates of anastomoses were 14.6, 18.5, 18.7% in MCAs group compared with 35.4, 36.9, 34% in hand-sewn group at 1, 3, and 6th month after RYHJ, respectively (*p* < 0.002) (Table 3).

**Table 2:** Postoperative complications in two groupsafer RYHJ

Groups	Postoperative complications						
	Stricture of BEA	Leakage of BEA	Leakage of EEA	Cholangitis	GI hemorrhage	Intussusception	Total
MCAs	0	0	1	1	3	1	6
Hand-sewn	2	2	0	0	2	1	7
*p*	0.486	0.486	>0.99	>0.99	>0.99	>0.99	0.61

*BEA* bilioenteric anastomoses, *EEA* enteroenteric anastomoses, *GI* gastrointestine

**Table 3:** Narrowing rate of anastomoses in two groups

Groups	Initial total ID (mm)	Final total ID (mm)	Narrowing rate (%)	*p*
PO 1 month				
MCAs	30.8 ± 4.32	26.3 ± 3.73	14.6	
Hand-sewn	44.93 ± 12.01	29.03 ± 7.4	35.4	0.002*
PO 3 month				
MCAs	35.88 ± 5.02	29.35 ± 5.18	18.5	
Hand-sewn	43.8 ± 9.5	28.05 ± 8.48	36.9	0.002*
PO 6 month				
MCAs	33.02 ± 6.72	26.26 ± 6.47	18.7	
Hand-sewn	39.57 ± 5.16	26.16 ± 3.68	34.0	0.002*

* *p* < 0.05, the difference between two groups is statistically significant

*PO* post-operation, *ID* interior diameter. Initial total ID was the average of total ID in subgroups which was measured before anastomotic construction. Final total ID was the average of total ID in subgroups which was measured at the end of each time point

### Histological analyses

**Fig. 5:**
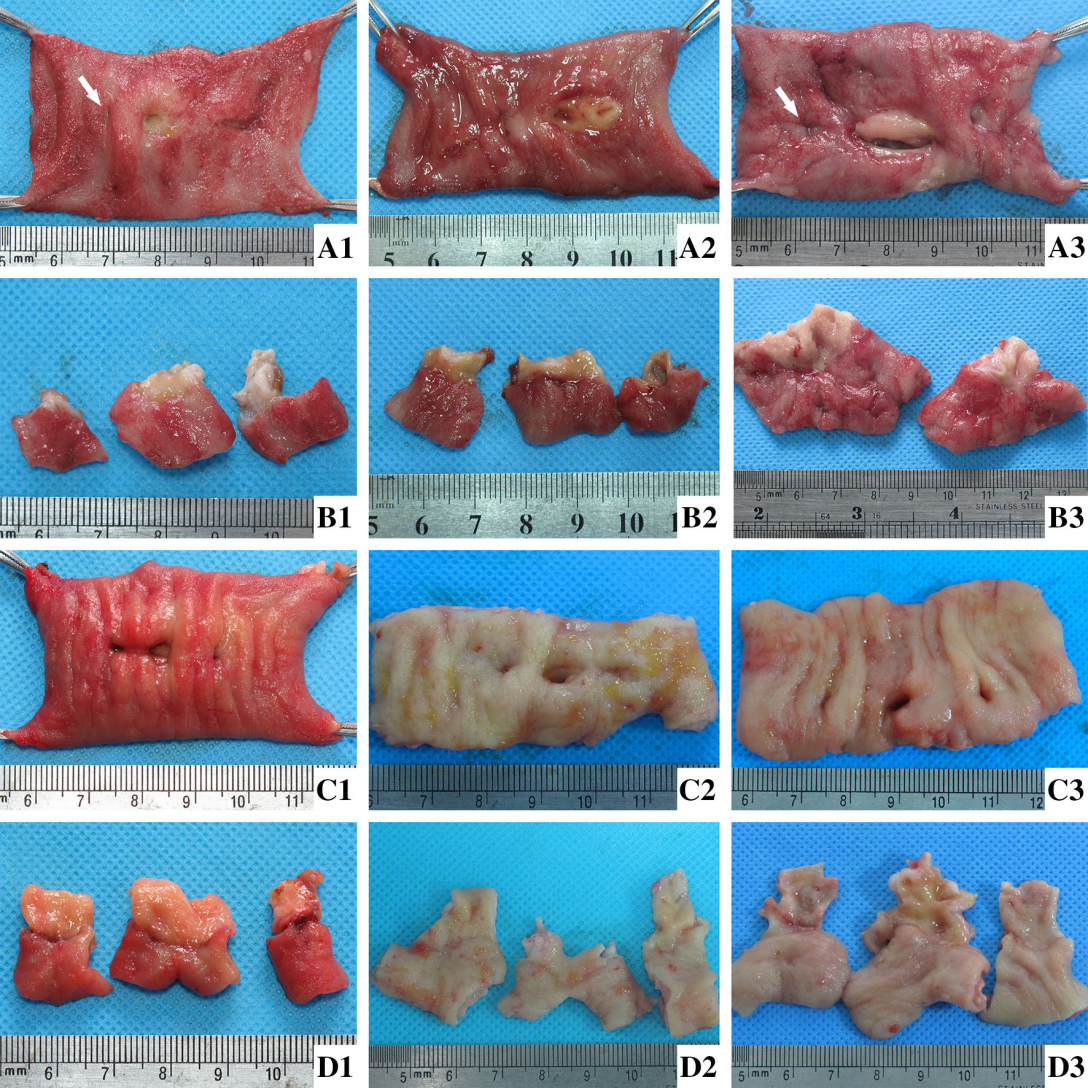
The gross appearance of anastomoses in two groups at 1, 3, and 6th month after RYHJ. **A1**–**A3** The anastomoses were exposed from the jejunal cavity at 1, 3, and 6th month after RYHJ in control group. The *white arrows* in **A1** and **A3** were sites where anastomotic stricture happened, and the right anastomosis was totally blocked at **A3**. **B1**–**B3** The anastomoses were incised along the long axis, the mucosal side of anastomoses was shown at 1, 3, and 6th month after RYHJ in control group. The view of right anastomosis was absent in **B3** because the lumen was blocked totally. **C1**–**C3** The anastomoses were exposed from the jejunal cavity at 1, 3, and 6th month after RYHJ in study group. **D1**–**D3** All the anastomoses were incised along the long axis, the mucosal side of anastomoses was shown at 1, 3, and 6th month after RYHJ in study group

At 1st month after RYHJ, the anastomotic mucosal surface in both groups was even and flat (Fig. 5B1, D1). However, the anastomoses at right heptic bile duct became stricture (Fig. 5A1) and remained biodegradable suture can be found at anastomoses in control group (Fig. 5B1). At 3rd month after RYHJ, the anastomoses healed better, and the suture in control group was absorbed completely (Fig. 5A2, B2). The anastomoses in both groups remained clear (Fig. 5A2, B2, C2, D2). At 6th month after RYHJ, one more anastomotic stricture happened at right hepatic bile duct in control group (Fig. 5A3, B3), but all anastomoses in study group kept clear and smooth (Fig. 5C3, D3).

**Fig. 6:**
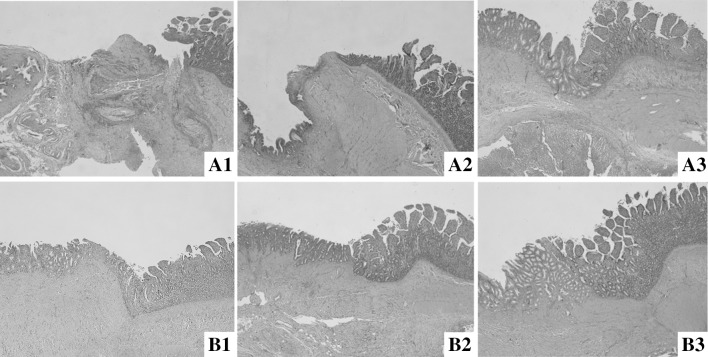
Histologic section of the anastomotic sites between two groups (HE-stain, ×40). **A1**–**A3** were images of the control group at 1, 3, and 6th month after RYHJ. **B1**–**B3** were images of study group at 1, 3, and 6th month after RYHJ

At the 1st month after RYHJ, displacement and poor alignment of tissue layers combined with severe inflammation were found in control group (Fig. 6A1). However, the tissue layers connected precisely with milder inflammation can be observed in study group (Fig. 6B1). At the 3rd month after RYHJ, in control group, the infiltration of local inflammative cells decreased with absorbing of the suture at anastomoses. However, the junction between the bile duct and jejunum protruded into jejunal lumen, and the reparation and reepithelialization of the wound did not finish yet (Fig. 6A2). In study group, the wound healed well with precise connection of layers (Fig. 6B2). At the 6th month after RYHJ, the inflammation in both groups was diminished. While the healing in control group was improved (Fig. 6A3), the preciser alignment of layers can be found in study group (Fig. 6B3).

The scores for bridging and the inflammatory reaction between the groups at each time point are detailed in Table 4. Histologic healing in study group was better than in control group and statistical significance can be seen between two groups at 1st month (*p* = 0.005) and 3rd month (*p* = 0.029), but not at 6th month (*p* > 0.05) after RYHJ.

**Table 4:** Histological evaluation of anastomotic healing in two groups

Groups	Bridging	Inflammatory reaction	Total
PO 1 month			
MCAs	7.75 ± 0.96	9.25 ± 0.96	17.0 ± 1.83
Hand-sewn	2.33 ± 1.53	7.0 ± 1.0	9.33 ± 2.52
*p*	0.002*	0.029*	0.005*
PO 3 month			
MCAs	8.8 ± 0.45	11.2 ± 0.84	20.0 ± 1.22
Hand-sewn	6.8 ± 0.84	10.0 ± 1.58	16.8 ± 2.39
*p*	0.002*	0.172	0.029*
PO 6 month			
MCAs	9.0 ± 0.00	11.2 ± 0.75	20.2 ± 0.75
Hand-sewn	8.33 ± 0.82	10.3 ± 0.82	18.7 ± 1.5
*p*	0.102	0.096	0.064

* *p* < 0.05, the difference between two groups is statistically significant

*PO* post-operation

### Tensile strength

**Fig. 7:**
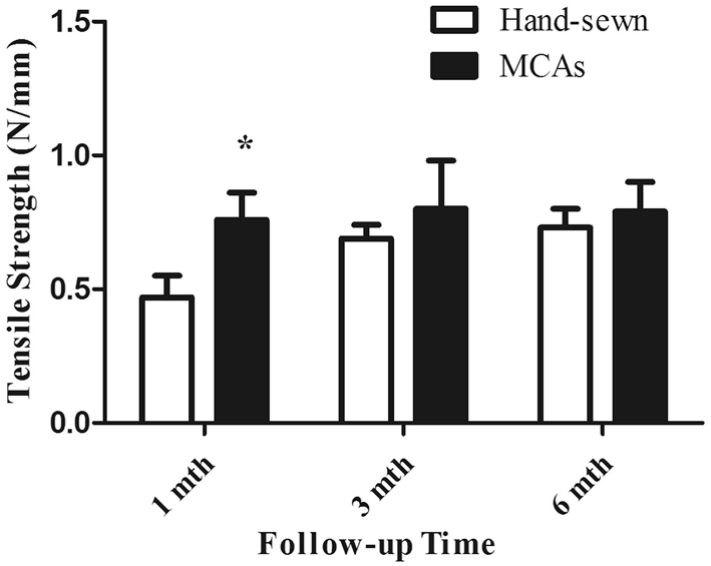
Bar graph (mean, SD) of anastomotic tensile strength testing in two groups (*Asterisk* statistically significance)

The tensile strength of anastomoses at different time points between the groups is compared in Fig. 7. The values were similar at 1, 3, and 6th month time point in MCAs group. However, the values in hand-sewn group increased gradually with extended follow-up period, and reached to the highest level similar to MCAs group.

### Content of hydroxyproline

**Fig. 8:**
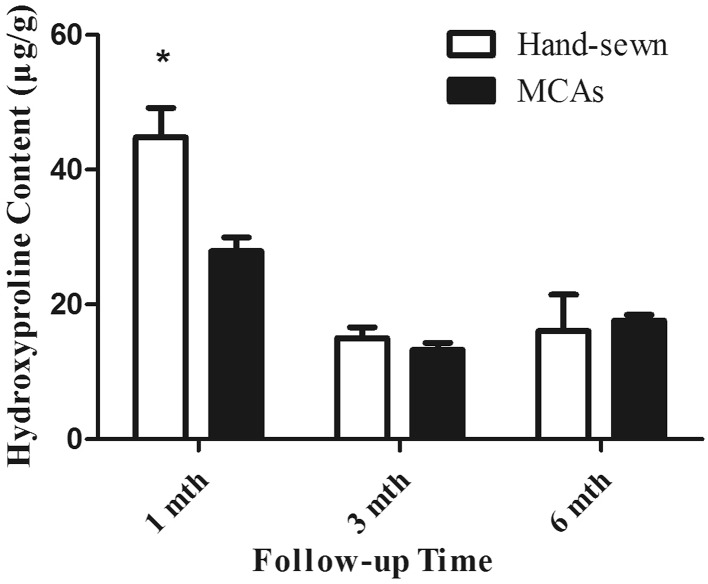
Bar graph (mean, SD) of anastomotic hydroxyproline content testing in two groups (*Asterisk* statistically significance)

Hydroxyproline content of anastomoses in two groups at different time points between the groups is shown in Fig. 8. The hydroxyproline in MCAs group was much lower than in hand-sewn group at 1st month after RYHJ. The values in both groups decreased at 3rd month and keep relatively consistent until the end of 6th month.

## Discussion

Application advanced anastomats is a symbol of modern surgical development. It makes surgery performed more efficiently, standardly, and consistently, and accelerates the development of minimal invasive surgery by laparoscope or Da Vinci^®^ surgical system. However, RYHJ remains to be open, conventional, hand-sewn procedure because no anastomat is available for such complicate and high-risk surgery. Individual disparity of lesions, small caliber of extrahepatic bile duct and multiple anastomoses construction make all clinical routine used anastomats unfit for RYHJ.

Magnetic compression anastomats, which have the character of auto-attraction, can be constructed simple and small, turn out to be the potential choice for performing RYHJ. Because these multiple bile duct stumps need to be reconstructed in RYHJ, the introduced magnetic anastomats should always be kept independent until be expelled out of the body. Otherwise, the adjacent anastomats would attract together, and following bile leakage or enteric leakage would happen. Since paramagnetic material can change the magnetic field distribution around the magnets, cylinder-shaped cast iron layer was milled as static magnetic field shielders, coated magnetic ring cores to keep the non-attractive between the magnetic anastomats. In our experiment, all well-coupled anastomats remained independent in anastomotic situ and throughout the expelling process in intestine.

The leakage and stricture are two most common anastomotic specific postoperative complications of RYHJ. The suture, although with advanced production, is still considered to be the most important factor to activate collagen enzyme and stir foreign-body-related inflammation, therefore inhibits the healing process and destroys the surgical results [32]. We designed the magnetic anastomats, which can automatically drop off from anastomoses and be expelled out of the body, made the anastomoses the least foreign-body reaction throughout healing process to improve the outcome and reduce the morbidity of post-operative complications.

With the help of MCAs, anastomotic construction in RYHJ is transformed from traditional stich-by-stich suturing to three-step operation: purse-string suturing, anastomats introduction, and MCAs coupling. The shortened anastomotic time means less celiac exposure during surgery and lower risk for surgical infections [33]. In addition, the simplified and standardized surgical procedures imply that shorter learning curve and less operation experience are needed for surgeons with MCAs.

In our experiment, both incidence of bile leakage and anastomotic stricture in hand-sewn group were 11.1%, which is similar to reported 5.6–10% [34–36] of bile leakage and 5.3–10% [34, 37] of anastomotic stricture in human after RYHJ. However, there is no single case of anastomotic-specific complications happened in MCAs group except three inadvertent application faults, which are one case of magnet core assembling fault and two cases of anastomats coupling failure. At early stage of the study, a few anastomats were assembled coupling sides with homopolar side of two magnets by mistake. We used one uncorrectly assembled EE-MCA in dog unintended. It was really hard to notice this problem during anastomosis since the mother part and daughter part did not reject against each other too much with the interference of the iron shielders. The anastomosis failed and the anastomats resided at situ without expelling because the device did not compress the tissue tightly and completely. When we realized this problem and checked through all the anastomats, the similar accident has never happened again. Two cases of anastomats coupling failure happened in first five dogs by MCAs. While surgery team gained more experience on correct use MCAs and followed the rule of taking routine abdominal fluoroscopy before closing the abdomen to check the anastomats’ situation, the miscoupling accident was disappeared. Therefore, we think that all anastomat application-related complications can be eliminated through short-term training on how to correctly use MCA. Actually, the magnetic compression anastomosis is but more than an inverting suture pattern. Sequential healing from serosa to mucosa and accurate connection of anastomotic edge contribute to the superior alignment and bridging of tissue layers. Meanwhile, auto-drop off character of MCAs diminished the suture-related inflammatory cell penetration, foreign-body giant cell forming, excessive granulomatous reaction, and fibroplasia at anastomoses. All these characters promote wound revascularization, granulation, and epithelialization in MCAs group.

We were once afraid that there was no enough strength to keep the anastomotic integrity in MCAs group at early stage when anastomat dropped off from anastomosis. However, there was no single case of anastomotic leakage happened in successfully coupled MCAs group. The results of breaking strength test showed that the MCAs group completed histologic healing in 1 month, and the comparable healing condition took at least 3 months in hand-sewn group. The later hydroxyproline content test further illustrated that the wound healing and construction in MCAs group is much quicker than in hand-sewn group.

Besides mentioned above, other characters of magnamosis, such as no bile and liquor entericus, penetrate into the anastomoses while wound healing, magnetic influence on tissue physiological activity [38, 39], may result in the better wound healing process. The related mechanism is an area worthy of further exploration.

To our best knowledge, it is first time to report performing multiple bilioenteric anastomosis by anastomats and first time to report constructing both bilioenteric and enteroenteric anastomoses in one type of anastomats. Although the results of the experiment are inspiring so far, the design and production of MCAs should be further optimized to improve the safety and convenience of clinical use. Everyone who use the anastomats should recognize the imaging features of coupling failure, which mostly are constant big gap or acute angle formed between two parts of the anastomats, long detained anastomats and multiple attracted anastomats at anastomoses without expelling. Relevant principle for magnetic compression anastomosis, such as routine fluoroscopy to check out inadvertent miscoupling after surgery, or keeping away from high-intensity magnetic field to prevent possible accident before MCAs are expelled out of the body, should be included in the operation specification.

The first generation MCAs have been successfully used for choledojejunostomy in more than forty cases in our center, and the following long-term follow-up results will come out later.

## Conclusion

The new designed MCAs can simplify the anastomotic construction in RYHJ in obstructive jaundice dog models. Owing to preciser alignment of tissue layers and absent of suture-stirred foreign-body reaction, the anastomotic healing is better and faster in MCAs group. The MCAs show an exciting perspective for clinical use.

## Electronic supplementary material

Below is the link to the electronic supplementary material.

Supplementary material 1 (DOCX 12 kb)

Supplementary material 2 (JPEG 3017 kb)

Supplementary material 3 (JPEG 1069 kb)
