# Supplementary material for: Advanced Roux-en-Y hepaticojejunostomy with magnetic compressive anastomats in obstructive jaundice dog models
Source: Surg Endosc. 2017 Aug 4;32(2):779–89. doi: 10.1007/s00464-017-5740-5 (PMC5772124; doi:10.1007/s00464-017-5740-5)
Supplement: Supplementary file 1 — Supplementary material 1 (DOCX 12 kb) [file 464_2017_5740_MOESM1_ESM.docx]

**Supplementary material**

**Fig. 9 Abdomen X-ray of miscoupled MCAs in study group**

A and B were BE-MCA miscoupling which happened at 5th day after HJ (A. anteroposterior film; B. lateral film). C. EE-MCA miscoupling happened at 5th day after HJ, acute angle was formed between two parts of the anastomat. The black arrows in B and C indicate miscoupled anastomats.

**Fig. 10 BE-MCAs retained at anastomoses at 1^st^ month after HJ**

A.Three BE-MCAs were attracted together and bile sludge formed around the anastomats after cut the Roux-en jejunal loop laterally. B. Intact right and left anastomoses have formed after removing the anastomats from the middle one. The middle anastomosis was not fully constructed yet because of miscoupling.

**Table 5．Anastomat application faults in study group**

| **The type of faults** | **Cases** | **Anastomat type** | | **The date find the faults(PO)** | | | **Symptoms** | **Treatments** | **Results** |
| --- | --- | --- | --- | --- | --- | --- | --- | --- | --- |
| Magnet core assembling fault | 1 | | EE-MCA | | 3^rd^ day | Lose appetite  Weakness | | Abdominal exploration | Dead |
| Anastomats coupling failure | 2 | | EE-MCA | | 5^th^ day | -- | | Abdominal exploration | Alive |
|  |  |  | BE-MCA | | 5^th^ day | Poor appetite  Emaciation  Jaundice | | -- | Cholangitis |

*PO* post-operative
