# Supplementary figures and images for: Advanced Roux-en-Y hepaticojejunostomy with magnetic compressive anastomats in obstructive jaundice dog models
Source: Surg Endosc. 2017 Aug 4;32(2):779–89. doi: 10.1007/s00464-017-5740-5 (PMC5772124; doi:10.1007/s00464-017-5740-5)

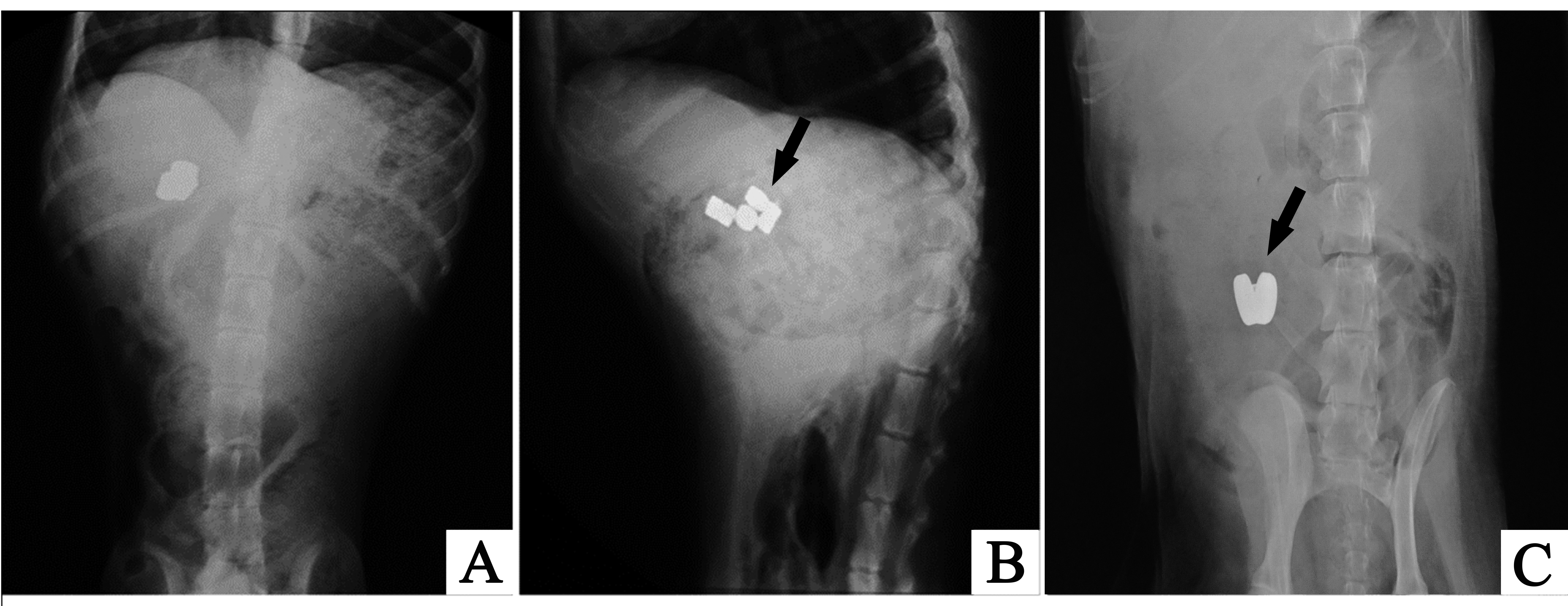

Supplement: Supplementary file 2 — Supplementary material 2 (JPEG 3017 kb) [file 464_2017_5740_MOESM2_ESM.jpg]

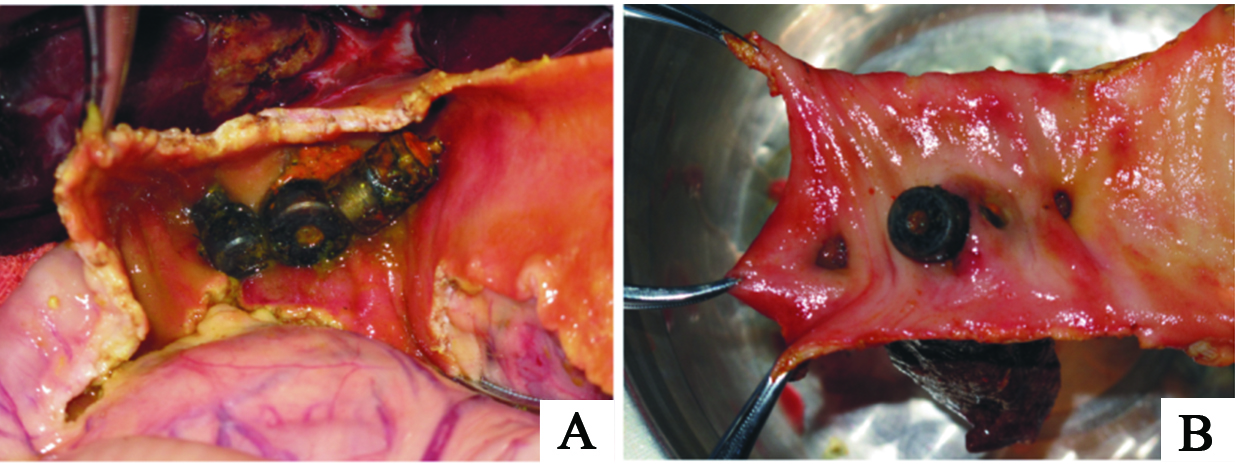

Supplement: Supplementary file 3 — Supplementary material 3 (JPEG 1069 kb) [file 464_2017_5740_MOESM3_ESM.jpg]
